# Fine-mapping within eQTL credible intervals by expression CROP-seq

**DOI:** 10.1093/biomethods/bpaa008

**Published:** 2020-03-28

**Authors:** Yidan Pan, Ruoyu Tian, Ciaran Lee, Gang Bao, Greg Gibson

**Affiliations:** b1 Systems, Synthetic, and Physical Biology, Rice University, Houston, TX, USA; b2 Department of Bioengineering, Rice University, Houston, TX, USA; b3 Center for Integrative Genomics, Georgia Institute of Technology, Atlanta, GA, USA; b4 APC Microbiome Ireland, University College, Cork, Ireland

## Abstract

The majority of genome-wide association study (GWAS)-identified SNPs are located in noncoding regions of genes and are likely to influence disease risk and phenotypes by affecting gene expression. Since credible intervals responsible for genome-wide associations typically consist of ≥100 variants with similar statistical support, experimental methods are needed to fine map causal variants. We report here a moderate-throughput approach to identifying regulatory GWAS variants, expression CROP-seq, which consists of multiplex CRISPR-Cas9 genome editing combined with single-cell RNAseq to measure perturbation in transcript abundance. Mutations were induced in the HL60/S4 myeloid cell line nearby 57 SNPs in three genes, two of which, rs2251039 and rs35675666, significantly altered *CISD1* and *PARK7* expression, respectively, with strong replication and validation in single-cell clones. The sites overlap with chromatin accessibility peaks and define causal variants for inflammatory bowel disease at the two loci. This relatively inexpensive approach should be scalable for broad surveys and is also implementable for the fine mapping of individual genes.

## Introduction

The majority of genome-wide association study (GWAS)-identified SNPs are located in noncoding regions of genes and are likely to influence disease risk and phenotypes by affecting gene expression [1]. Fine mapping of causal variants responsible for these signals is important for understanding which genes mediate phenotypic variation, dissecting mechanisms of action, assembling regulatory networks, and designing therapeutic interventions. It is recognized increasingly that GWAS peaks have a complex structure, the resolution of which is limited by linkage disequilibrium (LD) and the presence of multiple independent signals at many loci [2–4]. Since GWAS peaks often overlap with expression quantitative trait loci (eQTL) signals, namely associations with gene expression, transcription-based experimental screening approaches can be used to prioritize likely causal variants within credible intervals that contain ≥100 polymorphisms. Two classes of approach have been reported, Clustered Regularly Interspaced Short Palindromic Repeats-Cas9CRISPR-Cas9) genome editing [5], and massively parallel reporter assays [6], but have not been developed to systematically scan across the regulatory element(s) of a target gene.

Previous high-throughput CRISPR-based approaches to dissecting the impact of noncoding DNA have focused on defining cis-acting regulatory elements, rather than allelic effects of polymorphisms. They have generally utilized selection strategies followed by sequencing of barcodes from bulk cellular populations, assaying for enrichment or depletion of guide RNAs (gRNAs) targeting elements that are required for gene expression. In this way, Sanjana *et al*. [7] surveyed 700 kb around the *NF1*, *NF2*, and *CUL3* loci by selecting for resistance to inhibition of BRAF in a melanoma cell line when transcription of the genes is reduced, and Rajagopal *et al*. [8] tiled 40 kb around four genes into which they had inserted a green fluorescence protein marker to select for gene expression.

Extending this approach genome-wide, two groups have surveyed function of the majority of binding sites for p53 and estrogen receptor-α in the context of oncogene-induced senescence in a breast cancer cell line [9], and for FOXA1 and CTCF mediation of target gene activity in breast and prostate cancer cell growth [10]. These experiments define enhancer elements required for essential gene function, and incidental findings related to the existence of polymorphisms in some elements are reported, but they do not provide a mechanism to systematically scan candidate SNPs in credible intervals.

**Figure 1:**
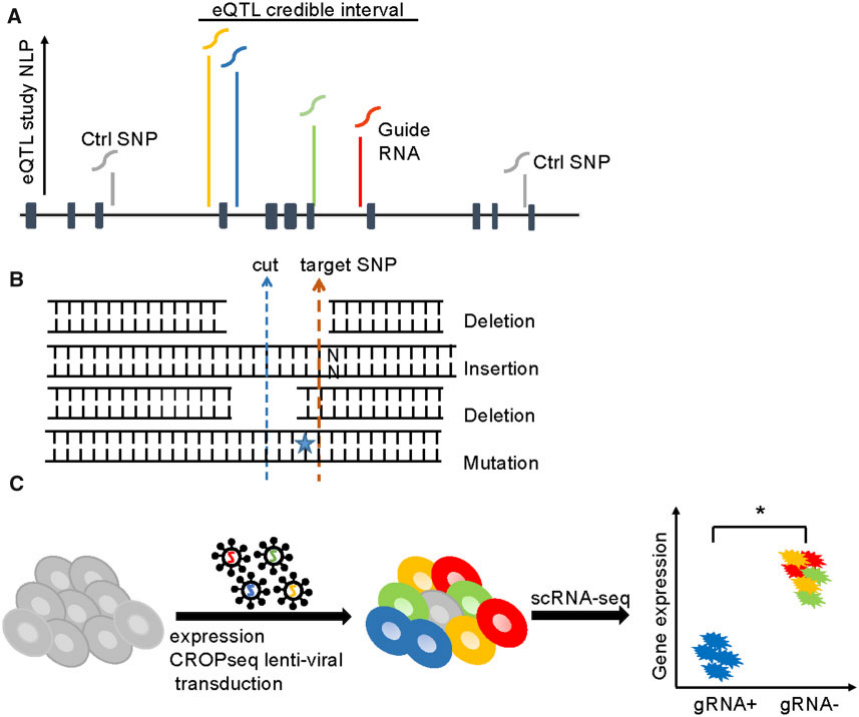
Experimental design of expression CROP-seq screening of eSNPs. (**A**) SNPs were selected with various eQTL *P*-values from one or two credible intervals for each eGene. Additional SNPs in low LD with the credible interval were selected as control SNPs. Each SNP was targeted by a single gRNA with minimal predicted off-target effect. The horizontal black line represents a hypothetical locus with exons indicated by solid blocks. (**B**) The Cas9 editing site may be a few bases away from the targeted SNP and can introduce four possible genetic alterations: deletion of both the cutting site and target SNP; insertion; deletion of only the cutting site; and mutation of the target SNP. (**C**) Pooled CROP-seq lentiviral libraries with 67 gRNA were transduced into the HL60/S4 cell line. Most cells were transduced with a single gRNA. Red, green, yellow, and blue represent four different gRNAs. A few cells have zero (gray cell) or multiple gRNAs. After 10X single-cell RNA-seq identified the gRNA of each cell, differential expression of the linked transcript is evaluated between cells with the gRNA relative to cells with all other gRNAs.

Here we report an adaptation of the CROP-seq (CRISPR droplet sequencing) protocol [11], for regulatory fine-mapping. CROP-seq involves multiplex CRISPR-Cas9 transfection of a cell line with dozens to hundreds of gRNAs targeting different genes, followed by single-cell RNAseq (scRNAseq) transcriptome profiling to monitor the consequences of inferred editing of the target gene. Even though not all cells are edited, the ability to detect which gRNA was present in each sequenced cell allows quantitative comparison of the effect of loss of function of the gene. In expression CROP-seq, we instead transfect dozens of gRNAs targeting different eSNPs in a credible regulatory interval and use the scRNAseq to monitor abnormal expression of linked transcripts (Fig. 1). Micro-deletion or mutation of the SNP in hundred or more cells provides sufficient power to detect up- or downregulation of expression consistent with most eQTL effect sizes. In a single experiment, we screened 57 SNPs in eQTL intervals of three genes associated with inflammatory bowel disease (*CISD1*, *PARK7*, and *DAP*), and showed, with replication and subsequent validation, that in two cases a single SNP located within an open chromatin peak is likely responsible for the genetic association.

## Materials and methods

### gRNA design and cloning

Approximately 20 SNPs were chosen for each gene based on prior eQTL mapping in peripheral blood mononuclear cells (PBMC) [4], along with five positive controls targeting the coding regions of the essential genes, *TUBB* and *RUNX1*, three negative controls that have no perfect target in the human genome, and one non-SNP targeting control. Each SNP was targeted for mutation, micro-deletion, or micro-insertion, by one single gRNA predicted *in silico* with COSMID software [12] to have a minimal likelihood of inducing off-target effects. The chromosomal position of each SNP and the flanking sequences were obtained from dbSNP [13]. All 19-base sequences followed by the correct *Streptococcus pyogenes* (*S. pyogenes)* Cas9 protospacer adjacent motif (PAM) sequence (NGG) inside the window were screened. gRNAs with GC rate >80% or <40% were filtered out to ensure better cutting performance. The gRNAs with the shortest distance from the cut site to targeted SNP (in most cases <10 bases) and minimal predicted off-target effects were used in our study. All selected gRNAs (listed in Supplementary Table S1) have only one perfect match to the whole reference genome, and negative controls had no perfect match in the human genome.

The CROPseq-Guide-Puro plasmid [11] (Addgene, Watertown MA, catalog number #86708, originally from Christoph Bock’s lab) was digested by Esp3I (NEB, R0734S). For each designed gRNA sequence, a pair of annealed oligos was cloned into the vector before the gRNA scaffold and after the U6 promoter. Clones were pooled for Maxi-prep (Qiagen, Hilden Germany, catalog number #12165) following the manufacturer’s protocol. The gRNA distribution in the plasmid prep was validated by next-generation sequencing.

In order to estimate the nature and rate of editing, single-cell clones were generated from the same cell population that was transduced by the two lentivirus vectors in preparation for scRNAseq. Integrated gRNA sequences of each single-cell clone were identified by amplifying a 300–400 bp fragment surrounding the relevant target SNP from 73 single-cell clones representing one of the 19 individual gRNAs. Next-generation sequencing was used to characterize the edited sequences. Supplementary Table S2 reports the exact edit in each clone and shows that 92% of the cells contained at least one edited allele, with 29% showing a single edited allele. The remainder either had two different edits, or only a single edit (implying biallelic editing, or that the alternate allele was not amplified). Additional columns show whether the target SNP was disrupted by a mutation within 3 bp of the target (67% of all clones) or whether the target SNP was directly disrupted (46%).

### CROP-seq lentivirus library construction and transfection

Lentivirus production from lentiviral vectors CROPseq-Guide-Puro and lentiCas9-Blast [14] (Addgene, 52962) and was performed following Addgene’s standard lentivirus production protocol using the Lenti-X 293 T cell line (Takara, Kusatsu Japan, catalog number #632180). LentiCRISPRv2GFP [15] (Addgene, catalog number #82416) was used as the reporter in each transfection. Lentivirus was pelleted by using L-90K ultracentrifuge (with SW32-Ti rotor, 25 000 rpm for 1.5 h at 4°) and dissolved in 100 μl 1×PBS.

Spinfection of HL60/S4 was performed according to the protocol from Feng Zhang lab [16]. Cells were seeded in a 24-well plate at a density of 1 × 10^6^/ml with 5 μg/ml of polybrene (EMD Millipore, Burlington MA, catalog number TR-1003-G). Up to 10 microliter (μl) of concentrated lentivirus was then added to each well, and cells were centrifuged at 1200 × g for 1.5 h at 33°C. HL60/S4 cells were first transduced by CROPseq-Guide-Puro lentivirus. Twenty-four hours after spinfection, cells were replated at a density of 5 × 10^5^/ml with 2 μg/ml puromycin (SigmaAldrich, P8833) selection for 8 days or until no viable cells were observed. Cell viability was monitored every 24 h, the media was changed every 48 h, and cell density was maintained under 1 × 10^6^/ml. The multiplicity of infection (MOI) was calculated, and the group with the least nonzero MOI was marked as HL60/S4-PuroR and used for downstream experiments for achieving optimal single gRNA assignment in the cell population.

After 3 days of recovery in regular culture media, HL60/S4-PuroR was transduced by lentiCas9-Blast lentivirus using spinfection with the same protocol as the CROPseq-Guide-Puro lentivirus transduction. Twenty-four hours after spinfection, cells were plated at a density of 5 × 10^5^/ml with 10 microgram (μg) / ml blasticidin (Research Products International, Mt Prospect IL, catalog number B12200) selection for 7 days. After 3 days of recovery, cells were further selected by dual drug selection for 3 days (1 μg/ml puromycin and 5 μg/ml blasticidin) to remove residual nonpuroR-blastR cells. Cells were then cultured in normal media for 10 days for global gene expression recovery.

### Single-cell RNA sequencing and data processing

Single-cell RNA sequencing libraries were prepared using 10X Genomics (Pleasanton, CA, USA) Chromium single-cell 3′ reagent kit V2 (PN-120267) and V3 chemistry (PN-1000092) with fresh cells for Replicate 1 and Replicate 2, respectively. The average cDNA library size was 484 bp and 505 bp for Replicate 1 and Replicate 2, respectively. Sequencing was performed on an Illumina NextSeq 550 system in high-output mode, generating paired-end libraries (28 bp for read1 and 98 bp for read2). Raw sequence data were first de-multiplexed from BCL files into FASTQ files by using “cellranger mkfastq”, with 10X Cell Ranger software. The human reference genome (hg38) was supplemented with 67 gRNA artificial chromosomes, each of which includes 241 bp U6 promoter sequences, 8 bp gap sequences between U6 promoter and gRNA, 20 bp gRNA sequences and 261 bp backbone sequences downstream of the gRNA. This 67 gRNA extended hg38 was indexed by “cellranger mkref” with extension “.fa” and “.gtf” files as input. Single-cell gene counts were generated by “cellranger count” by aligning reads to the extended hg38 by STAR aligner [17] with default settings. The estimated total number of cells detected was 8671 and 10 087 for Replicate 1 and Replicate 2, respectively. The average total sequencing read depth per cell were 58 413 and 45 636 for Replicate 1 and Replicate 2, respectively.

**Figure 2:**
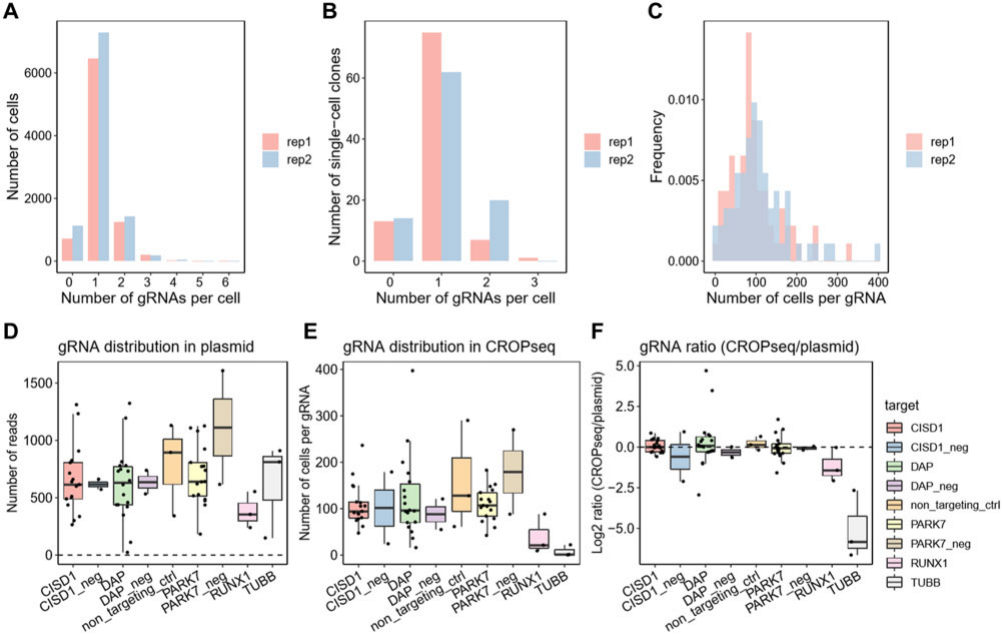
Guide RNA distributions. (**A**) The distribution of number of gRNA per cell detected from scRNAseq. (**B**) Similar distributions were observed for amplified gDNA insertions in 96 single-cell clones. (**C**) Histogram showing the number of cells containing each gRNA in the two replicates. (**D**) Raw read counts of each gRNA in the pooled library with respect to the candidate eSNPs or negative controls. (**E**) Number of cells assigned to each gRNA in scRNAseq shows a similar profile. (**F**) Log2 ratio of normalized RNA to DNA implying no deviation from expected equivalence, except for positive controls in the essential genes *RUNX1* and *TUBB*.

Each cell was distinguished by a cell barcode and a gRNA sequence, and in the majority of cases, a single gRNA was uniquely assigned to each cell (Fig. 2A). To confirm that the scRNAseq profiles adequately represent rates of lentiviral transformation, we amplified and sequenced the integrated gRNA sequence from 96 single-cell derived CRISPR-Cas9 clones, observing a similar distribution of gRNAs (Fig. 2B).

The count of unique molecular identifiers (UMIs) for each gRNA per cell was quantified by “cellranger count.” The gRNA-cell expression matrix was extracted from the cell-gene expression matrix. Only cells with a single gRNA expressed were included in the downstream analysis. If the gRNA UMI count was >0, it was coded as 1, otherwise coded as 0. This updated gRNA-cell identity matrix was appended to the cell gene expression matrix, providing the gRNA assignment for each uniquely assigned cell.

### Expression data quality control and normalization

The R package Seurat V3.0 [18] downloaded from https://github.com/satijalab/seurat was used for scRNAseq expression data processing and analysis. Low-quality cells with <200 genes expressed as well as lowly expressed genes detected in fewer than six cells were filtered out. Next, cells that had between 2000 and 7000 expressed UMIs and cells having <25% mitochondrial counts were retained. After quality control, 8192 cells and 16 372 genes were kept for Replicate 1, and 8921 cells and 16 407 genes were kept for Replicate 2. Then gene expression measurements were normalized by dividing by the total UMI counts and multiplying by the scaling factor 10 000, and transformed to logarithm base 2.

Linear dimensional reduction principal component analysis (PCA) was first performed with the top 2000 identified highly variable genes and default settings in Seurat [18]. The “JackStraw” function implemented in Seurat was used to determine the significant PCs. Nonlinear dimensional reduction by uniform manifold approximation and projection (UMAP) [19] was then performed based on the top 20 significant PCs with default settings. Each cell was assigned a score summarizing expression of G2/M and S phase gene markers implemented in Seurat package, and thereby classified into either G2M, S, or G1 phase according to its cell cycle score. The UMAP projection in Supplementary Fig. S1 suggests some clustering of cells by cycle identity (f) which also correlates with read depth (e) and number of detected genes (d). However, individual transcripts do not cluster with respect to these properties (a–c).

### Hypothesis testing

While more complex models, e.g., cell cycle fitting, or matching cells according to UMI count, were considered, they did not change the conclusions. We, therefore, report the simplest statistical approach to hypothesis testing. Each of the candidate eSNPs from one credible interval was fit with the normalized expression of its respective target eGene. Univariate linear regression was performed with “lm” function in R. Student’s *t*-test was conducted to test the null hypothesis that the coefficient of eSNP equals to zero in the regression. Bonferroni correction was applied for the simultaneously performed independent *t*-tests within each locus, namely alpha = 0.05 divided by 20 tests per gene for a gene-wise nominal adjusted critical value of 0.0025.

### Validation in CRISPR edited cells with single gRNAs

We further validated the effects of the two identified eSNPs on expression of the target genes *CISD1* and *PARK7* with two single gRNA approaches. First, HL60/S4 cells were transduced by lentiCas9-Blast and CROPseq-Guide-Puro lentivirus with the same gRNAs targeting rs2251039, rs35675666, or a negative control, using the same protocol as described before but as individual gRNAs in three separate transfection experiments. DNA was extracted from the bulk edited cells, the targeted regions were amplified by PCR, and genotypes were assessed by Sanger Sequencing (Eurofins Genomics) using the Synthego ICE strategy [20]. Supplementary Table S4 shows the proportions of most commonly edited alleles at the two loci, accounting for 87% of the rs2251039 and 79% of the rs3567566 edits. In both cases, the bulk of the edits are micro-insertions or micro-deletions (indels) indels adjacent to the SNP. Transcript abundance in duplicate bulk RNA extracts was estimated by real-time quantitative reverse transcription-polymerase chain reaction (qRT-PCR).

Second, these bulk edited cell suspensions were single-cell sorted and seeded into 96-well plates with standard culture media and cultured for 14 days. Half of the cells for each single-cell clone were taken at Day 7 for genotyping using next-generation sequencing of the targeted region. From these, a set of single-cell clones that expanded successfully and had the SNP removed/affected were chosen for qRT-PCR, including seven rs22510396 gRNA-edited clones, six rs35675666 gRNA-edited clones, and four clones edited with a nontargeting negative control gRNA. The sequences of the targeted alleles are shown in Supplementary Table S5. RNA from each selected single-cell clone was extracted using a RNeasy Mini Kit (Qiagen, cat. no. 74104) and reverse transcribed with iScript™ cDNA Synthesis Kit (Biorad, cat. no. 1708891) following standard protocols. Cycle thresholds for *CISD1*, *PARK7*, *GAPDH*, and *ACTB* were quantified by qRT-PCR with three technical replicates. The 2^−ΔΔCt^ method was used to analyze the qRT-PCR results, in which gene expression in cells with gRNA targeting *CISD1* or *PARK7* was normalized by the average of corresponding expressions in negative controls as well as the average of two housekeeping genes. Similar results were obtained with each single control gene.

## Results

Multiplex CRISPR-Cas9 editing of myeloid HL60/S4 cells, followed by single-cell RNA sequencing (scRNAseq), was used to monitor the impact of candidate regulatory SNP disruption on gene expression of three genes in a single experiment. We screened 57 candidate SNPs along with 10 control SNPs, using lentiviral transfection of a single-cell clone of the HL60/S4 myeloid human cell line. Approximately 20 SNPs were chosen for each gene based on prior eQTL mapping in PBMC [4], along with five positive controls targeting the coding regions of the essential genes *TUBB* and *RUNX* [21], three negative controls that have no perfect target in the human genome, and one non-SNP targeting control. Each SNP was targeted for mutation, microdeletion or micro-insertion, by one gRNA predicted to have a minimal likelihood of inducing off-target effects [12]. Two lentiviral vectors were used to successively infect HL60/S4 cells, the first one encoding both the puromycin resistance gene, and a single gRNA (positioned such that transcripts containing the guide would be captured by RNAseq), the second encoding both a blasticidin resistance gene and the Cas9 enzyme. This design facilitates identification of which guide(s) from the pool of 67 guides in the transformation mix, each single cell has taken up.

Each cell was distinguished by a cell barcode and a gRNA sequence, and in the majority of cases a single gRNA was uniquely assigned to each cell (Fig. 2A). To confirm that the scRNAseq profiles adequately represent rates of lentiviral transformation, we amplified and sequenced the integrated gRNA sequence from 96 single-cell-derived CRISPR-Cas9 clones, observing a similar distribution of gRNAs (Fig. 2B). The distribution of cells per unique guide ranged from 10 to 550, with an average of 117.3 ± 66.5 cells, ensuring sufficient statistical power to detect eQTL with moderate to high effect sizes (Fig. 2B). Furthermore, each of the 67 gRNAs was evenly distributed in the transfection mix of cloned DNA plasmids. There were no significant fold changes in guide abundances in scRNAseq relative to DNA plasmid levels, with the exception of the essential genes *RUNX1* and *TUBB* (Fig. 2D and E). These results confirm the efficiency of Cas9-mediated editing and imply that disruption of the regulatory regions of the three target genes did not compromise cell viability.

**Figure 3:**
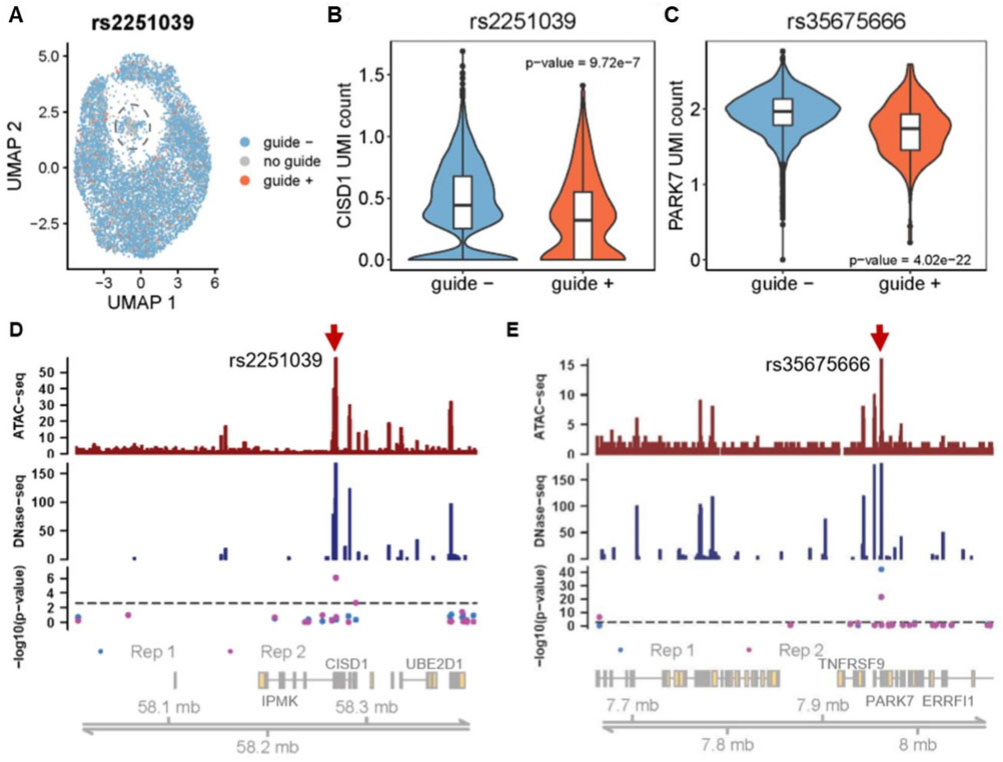
Identification of causal variants by expression CROP-seq. (**A**) Nonlinear dimensional reduction of 20PCs of single-cell transcriptome profiles by UMAP in Seurat (18). Cells are color coded as orange rs2251039 gRNA; blue gRNAs other than rs2251039; gray without any gRNA. Excluded cells with abnormally low number of UMI indicated by the dashed circle. (**B**, **C**) Violin plots show kernel density distributions of the expression of normalized log2 *CISD1* and *PARK7* UMI counts of cells with (+) or without (−) gRNAs targeting rs2251039 or rs35675666. Boxplots show the median, first and third quantiles of the data. (**D**, **E**) Chromatin accessibility of identified expression CROP-seq peaks for *CISD1* and *PARK7*. Top and middle histograms show HL60 ATAC-seq (GSM2083754) peaks and HL60 DNase-seq (ENCSR000ENU) peaks, respectively. The third panel shows the negative log10 *P*-value of student’s *t*-test statistic corresponding to the genomic location of the tested SNPs in two biological replicates, with the gene-wise Bonferroni adjusted *P* = 0.0025 threshold indicated by the dashed line. The bottom panel is a schematic of all gene transcripts annotated from the UCSC gene table (hg38). Red arrows point to the two inferred causal eSNPs in both replicates.

We characterized the transcriptional profiles of 6358 and 6974 single gRNA assigned cells in two biological replicates, with on average 58* *413 and 45 636 sequencing reads per cell. Cells with two or more gRNAs were excluded from the eQTL analysis. UMAP projection shows that the expression of *CISD1*, *DAP*, and *PARK7* was uniformly allocated among the clusters (Supplementary Fig. S1A–C). There was some clustering of the cells with respect to total number of reads and of UMI, which to some extent correlates with cell cycle stage (G1, S, or G2/M; Supplementary Fig. S1D–F), while a small number of low-transcript abundance cells were also excluded from further analysis. Figure 3A shows that cells with a single gRNA (e.g., targeting to rs2251039) (orange) and cells with gRNAs other than the one targeting to rs2251039 (blue) were also evenly distributed with respect to the clustering.

Univariate linear modeling was sufficient to resolve individual eSNP effects observed in two replicates of the experiment conducted several months apart. In the first replicate, two SNPs, one in *CISD1* (rs2251039) and one in *PARK7* (rs35675666) were identified as putatively causal (*P*∼10^−6^ and *P *<* *10^−20^; both with Bonferroni corrected *P *<* *0.0025). The same two SNPs replicated in the second experiment, at similar significance levels (Fig. 3B and C). Only two other nominally significant associations were observed, in a single replicate at *DAP* and a single replicate at *CISD1*. Moreover, we also examined if the knockout or mutation of targeted SNP would also influence the expression of adjacent genes. We tested the association between *CISD1* eSNPs with *IPMK* or *UBE2D1* expression, as well as between *PARK7* eSNPs and *ERRFI1* (*TNFRSF9* abundance was too low to assess), and between *DAP* eSNPs and *ANKRD33B* expression. None of the candidate eSNPs showed significant association with the nearby transcripts in either replicate (Supplementary Table S3).

Both of the significant SNPs were also among the most significant hits in the CAGE study that motivated sampling of the three genes [4]. Further evidence that they are likely causal is provided by the observation that they both lie under chromatin accessibility assay peaks. Figure 3D and E show the location of each assayed SNP at *CISD1* and *PARK7* relative to ATACseq and DNase-seq (ENCSR000ENU) profiles of HL60/S4 cells [22]. The majority of the nonsignificant expression CROP-seq SNPs lie between ATAC or DHS sites. Furthermore, rs2251039 is located just 28 bp upstream of the transcription start site of *CISD1* and is within a binding motif for the Bhlhe40 transcription factor, a known regulator of cytokine production in T-cells [23].

The function of both SNPs was validated using qRT-PCR on both bulk edited cells and in single-cell clones, with results illustrated in Supplementary Fig. S2. Bulk transfection of HL60/S4 cells with single guides resulted in downregulation of the associated *CISD1* and *PARK7* transcripts relative to cells transfected with nontargeting control gRNAs to a similar degree as inferred in the expression CROP-seq assays. More precise evidence for downregulation of gene expression after disruption of the targeted SNP was obtained by qRT-PCR of six single-cell clones containing indels in or adjacent to rs35675666 in *PARK7*, or seven single-cell clones containing indels in or adjacent to rs2251039 in *CISD1*. Relative to expression in four nontargeting control clones and normalized to the unaffected housekeeping genes *GAPDH* and *ACTB* with the 2^−ΔΔCt^ method [24], all targeted transcripts showed between 20% and 87% reduced abundance, with *P* < 0.005 for both genes.

## Discussion

Published genome-editing strategies for interrogating regulatory elements either utilize CRISPRi or CRISPRa to inhibit or activate transcription [25, 26], or rely on assays that select for essential gene function [7, 9, 10] or reporter gene expression [8]. Neither approach is suitable for systematically screening the function of each of the candidate SNPs in a credible interval of a typical gene. Massively parallel reporter assays have been used to this end more successfully. For example, Ryan Tewhey *et al*. [27] evaluated 32 373 variants at 3642 eQTL by inserting 180 bp oligonucleotides encompassing each SNP in front of a minimal promoter, finding 842 polymorphisms that drive reporter expression in a lymphoblast cell line at different levels. An even larger scale experiment by van Arensbergen *et al*. [28] surveyed 5.9 million variants, namely 57% of all known common variants in the human genome, by associating short DNA fragments with a barcode and assaying tag abundance in hepatic and erythroid cell lines. They identified over 30 000 candidate eSNPs, most cell type specific, and described enrichment with various chromatin features. Impressive as these studies are, there is always the caveat that enhancer activity outside normal chromatin context may not be accurate, and perusal of the SuRE database [28] suggests that many sites have large but nonsignificant effects since the majority of the cloned fragments do not drive expression. Hence, false-negative rates are not known, and complementary assays that systematically interrogate credible intervals in the same promoter context should also be informative.

Our approach is to directly measure expression of a gene after genome editing of a set of regulatory polymorphisms. Targeted reporter assays analyzing RNA from bulk preparations of clonal cell lines have low power to resolve typical eQTL effects that explain in the range of 10–20% of the variance of the target gene. The CROP-seq single-cell eQTL screening strategy gains power from the sequencing of thousands of cells in parallel. The effect size of rs2251039 in *CISD1* corresponds to a reduction of ∼0.5 standard deviation units due to gene editing, equivalent to an eQTL explaining between 5% and 10% of the variance (depending on the allele frequency and assumptions about whether one or both alleles are disrupted in the CROP-seq). The much larger rs35675666 effect at *PARK7* could correspond to a 3-times larger effect eQTL, or may reflect more efficient gene editing by the particular gRNA, which appears to create large deletions encompassing the SNP (Supplementary Table S2).

Interestingly, rs35675666 is located in the first intron of the *PARK7* transcript and is a GWAS SNP for ulcerative colitis (*P* = 5 × 10^−9^) and inflammatory bowel disease (*P* = 1 × 10^−15^) [29]. Although McCole [30] argued that *ERRF11* is a strong candidate gene in the interval due to the impact of ErbB receptor feedback inhibition on epithelial apoptosis and possibly barrier function, the absence of effect on *ERRF11* transcript abundance calls into question that inference and instead promotes *PARK7* as the likely causal gene. *PARK7* encodes a C56 peptidase family member that has been shown to function as a regulator of mitochondrial respiration and lysosomal function [31]. Autosomal recessive loss of function leads to early-onset Parkinson’s disease, and reduced expression may conceivably disrupt autophagy or oxidative stress sensing, both of which are implicated in ulcerative colitis [32].

In theory, expression CROP-seq should be powered to fine map eSNPs within credible intervals that explain just a few percent of the expression of a target gene. We were able to confirm the identity of autoimmune disease-associated GWAS variants in two loci, but did not detect the third eQTL or resolve the secondary associations that are nevertheless present at each of the loci we tested. Comprehensive fine mapping will often require ≥100 gRNAs per gene, but is well within the scope of the experimental pipeline described here. Limitations include the inability to target all SNPs due to absence of appropriate PAM sequences, reduced power for genes expressed at levels close to the limit of detection in scRNAseq, and appropriateness of the cell line(s) chosen for the assay. Replication is likely to be important for the confident identification of relatively small effect eSNPs, particularly given that cell passaging, mutation, and random variability during cell culture can affect the transcriptional background [33]. Future experiments may also use prime editing [34] to specifically replace one allele with the alternate allele, rather than inducing mutations at or near the site. Finally, we also show that integration with functional annotation data may help to validate inferred eSNPs and identify the likely transcription factors they bind. In all, our method facilitates the genetic screening of noncoding variants and the transcriptional interpretation of risk variants in the post-GWAS era.

## Supplementary Material

bpaa008_Supplementary_Data
